# Antipsychotic Use Pattern in Schizophrenia Outpatients: Correlates of Polypharmacy

**DOI:** 10.2174/1745017901713010092

**Published:** 2017-08-11

**Authors:** Esra Yazici, Ali S. Cilli, Ahmet B. Yazici, Hayriye Baysan, Mustafa Ince, Sukriye Bosgelmez, Serkan Bilgic, Betul Aslan, Atila Erol

**Affiliations:** 1 Department of Psychiatry, Sakarya University Medical Faculty Training and Research Hospital - Psychiatry Sakarya, Sakarya, Turkey; 2 Kocaeli Derince Training and Research Hospital - Psychiatry Kocaeli, Kocaeli, Turkey

## Abstract

**Background:**

This study investigates the antipsychotic use patterns of patients with schizophrenia and its correlations in their daily drug use patterns.

**Methods:**

Patients with schizophrenia who have regular records at two different community counselling centres (CCS) were included in the study. Information about their medications and sociodemographic data was recorded through face-to-face interviews and supporting information about their drug use patterns was obtained from their relatives/caregivers/nurse. The Clinical Global Impression Scale (severity of illness) and the General Assessment of Functionality scales were also administered.

**Results:**

Patients with schizophrenia used 2.0 ± 0.81 antipsychotics daily and 3.52 ± 2.55 pills (1–18). Seventy-one percent of the patients used two or more kinds of psychotropic drugs. The most frequently used antipsychotics were quetiapine, a second generation antipsychotic, and haloperidol, a typical antipsychotic. Clinical severity, regular visits to a CCS and use of depot antipsychotics were independent predictors for polypharmacy.

**Conclusion:**

The rate of polypharmacy use is high in Turkey. There are multiple risk factors related with polipharmacy. New studies should focus risk factors for preventing polypharmacy.

## INTRODUCTION

Both the type and the number of antipsychotics are increasing daily and new indications for antipsychotics are being added to the existing ones in updated guidelines. The treatment or/and the pharmacotherapy for schizophrenia are a dynamic process that can change over time. Although typical antipsychotics were widely used after the 1950s, second generation antipsychotics are currently common [1] The definition of polypharmacy varies according to the different authors and disciplines. In psychiatry, the use of two or more kinds of antipsychotics is defined as antipsychotic polypharmacy [2], while, in general, the use of two or more psychotropic drugs is defined as psychotropic polypharmacy [3].

Although, in clinical practice applications, there are several guidelines for the use of antipsychotics for schizophrenia, this varies in the real world. For example, antipsychotic polypharmacy (APP) is widely used in clinical practice. Despite the lack of robust evidence, the increased risk of side effects and the cost implications for this approach, APP is commonly used in schizophrenia. APP prevalence rates vary widely, from 4–92.2%, depending on study design, patient population, diagnosis and geographical region [4-6]. In previous studies conducted in Asia, the polypharmacy rates were similar or higher than Turkey results. A multicentre study conducted on schizophrenia patients in East Asia reported antipsychotic polypharmacy rates to be 45.7% [7]. Earleir studies in Asia reported antipsychotic polypharmacy rates of 90% in Japan [8] and 59% in Singapore [9]. On the other hand, in Europe, the rates were reported to be 43.9% in Germany [10], and in Spain, there was a 13.9% antipsychotic combination and 19.4% unspecified combinations [11]. A recently published retrospective cohort study (with 4,156 patients included in the study) in the United States reported that antipsychotic polypharmacy in patients with schizophrenia was 23.3% [12]. The polypharmacy rate in a Norwegian study of schizophrenic patients’ antipsychotics when they were discharged from an emergency ward was recorded and the polypharmacy rates were observed to be 35.6% and 24.9% when low potency first generation antipsychotics(FGAs) in doses below a 100 mg/day chlorpromazine (CPZ) equivalent dose were excluded [13]. In a one study in Nigeria polypharmacy psychotropic agents were reported as 92% for general psychiatric outpatients and 94% in schizophrenia patients [4].

The current data show that there may be some clinical benefits of antipsychotic polypharmacy in clinical challenges, such as with severe or treatment-resistant conditions. However, polypharmacy has harmful potential conclusions, such as an excessively high content of antipsychotic dosages [14, 15], extrapyramidal side-effects, cognitive impairment and drug interactions; it can also result in decreases in drug concentrations, thus insufficient treatment, and increasing cost, which may be associated with an increased risk of death [6, 16]. Using higher doses of antipsychotics leads to high dopamine D2 receptor antagonism and it is shown to be related to lower subjective well-being, a worse medication adherence and thereby a poor therapeutic outcome [17]

Chronic use of antipsychotics with a relatively high dose is also shown to be related with a worse outcome in long term recovery than the strategy of dose reduction or discontinuation [18]

It is well established that polypharmacy has harmful effects. It is not recommended and all psychiatrists undoubtedly know this information. Nonetheless, polypharmacy continues to exist in the real world and its use increases each year. Therefore, the question is ‘What is the reason for polypharmacy?’ There are several studies about the predictors of polypharmacy: the common associated factors are male gender, younger age and being single and unemployed. Other factors are primarily related to clinical severity and poor functionality of the patients, such as having severe psychopathology, residual psychotic symptoms, poor cognitive function, poor insight into the illness, additional psychiatric co-morbidity, in-patients of a psychiatric hospital, involuntary admission, more frequent admissions and the use of depot antipsychotics [6].

Polypharmacy continues to exist with increasing frequency and there is no clear answer regarding what to do about solving this problem. Consequently, there is still a need for updated natural/realistic data to understand the correlates as well as the severity of the condition. This study investigates the antipsychotic use patterns of patients with schizophrenia and its correlations in their daily drug use patterns.

## METHODS

### Sampling

This study was conducted in 2015 with 280 patients with schizophrenia whose treatment and rehabilitation programmes were provided by the Sakarya Community Counselling Centre (Sakarya TRSM) and the Derince Community Counselling Centre (Derince TRSM). Ethical approval was obtained from the Sakarya University Medical Sciences Ethical Committee. Written consent was obtained from all of the participants of the study.

The community counselling centres (CCS) in Turkey monitor patients with severe mental illness with schizophrenia, schizoaffective disorder or bipolar disorder as a primary diagnosis. Most of the patients are clinically stable outpatients and, in general, they come to the centre either daily, weekly and sometimes irregularly or they are visited in their home by the centre; this differs according to the patients’ clinical need, level of social support, environmental/economic status, *etc*. At the time when the study was conducted, Sakarya TRSM had 503 recorded patients with severe mental disease and Derince TRSM had 355 patients.

### Procedure

Records from previous six months were evaluated and patients were filtered in two stages. First, the ones with schizoaffective and bipolar disorders, the ones with comorbid substance-alcohol use disorders and the ones with organic disorders of the central nervous system, such as epilepsy, multiple sclerosis, *etc*., were excluded. Patients who had inconsistent or missing data were excluded. Patients with schizophrenia who used at least one type of antipsychotic were selected for the study. Patients whose data in the records was accurate, complete and available to be checked with the caregiver or nurse were asked to participate; only voluntary patients over age 18 were included in the study.

Second, the chief psychiatrists of both centres (SB and SB) checked the accuracy of the diagnosis of schizophrenia; the study was conducted on patients with schizophrenia according to DSM-V criteria. Patients who had been diagnosed with schizophrenia for at least one year and who had not been hospitalized during the last six months were accepted. Information regarding their medications and sociodemographic data was recorded through a face-to-face interview; supporting information regarding their drug use patterns was obtained from their relatives/caregivers/nurse. The Clinical Global Impression Scale (severity of illness) and the General Assessment of Functionality scales were also administered.

### Classification and Calculation of Equivalent Doses of Antipsychotics

The classification of antipsychotics as first generation and second generation was conducted according to Psychotropic Drug Directory [19] Doses of the antipsychotic were calculated as a chlorpromazine equivalent (**CPZeq**), according to a previous study conducted by Kroken *et al.* [13], which is reviewed and abstracted previous literature on a table of equivalents doses. It is known that sometimes the equivalent dosages under chlorpromazine 100 mg may not have the exact intention of their antipsychotic effects [1]. So two separate calculations were conducted for the antipsychotic polypharmacy: The first calculation included all of the antipsychotics in all dosages and in the second calculation included only the antipsychotics that were at higher dosages of chlorpromazine 100 mg equivalent dose. Plasma levels of antipsychotics including clozapine are not used in routine treatment follow-up procedure in CCS so plasma levels of drugs were not available in this study.

### Instruments

**Sociodemografical and Clinical Data Form: **This a form prepared to include sociodemografic and clinic data about the patients as the gender, age, education, marital status, occupation, family history, *etc*., and questions about the duration of illness, number of hospitalisations, suicide attempts, violence and treatment adherence.

**Clinical Global Impression Scale (for Severity) (CGI-S):** This scale is used to assess the severity, the improvement rate and the medication side effects of psychiatric disorders. The illness severity subscale was used in this study. The scale for the severity of illness refers to the clinician’s global impression of the patient; it is scored between 1 and 7. The scale scores rise as the severity of the illness increases [20].

**General Assessment of Functionality (GAF):** The GAF scale, which is structured in accordance with the DSM-IV, assesses the patient’s psychological, social and occupational functioning. The general framework of this scale uses a single measurement to help monitor clinical progress of an individual. It involves general rating of a person’s functioning at that moment or in the past and is done by a clinician who gives points between 1 and 100 [21].

**Statistical Analysis:** The data were analysed using the Statistical Package for the Social Sciences 17.0 (SPSS Inc., Chicago, IL, US). To compare the mean values of the linear variables, the Student’s *t*-test was used for the groups with a standard distribution and the Mann–Whitney *U*-test for those that did not have a standard distribution [22]. The chi-square test was used to compare categorical variables [23]. The Pearson’s correlation analysis was used for correlation analyses and the linear regression analysis for identifying the predictors [24].

## RESULTS

Finally, 149 schizophrenia patients from Sakarya TRSM and 131 from Derince TRSM (total 280 patients) who had regular records at the respective community counselling centres over the previous six months were included in the study.

The mean age of the patients was 40.9 ± 9.9 (19–70) and 66.3% of the patients were male. The sociodemographic and clinical properties of the patients are provided in (Table **1**).

### Kind, number and dosages of antipsychotics

**Fig. (1):**
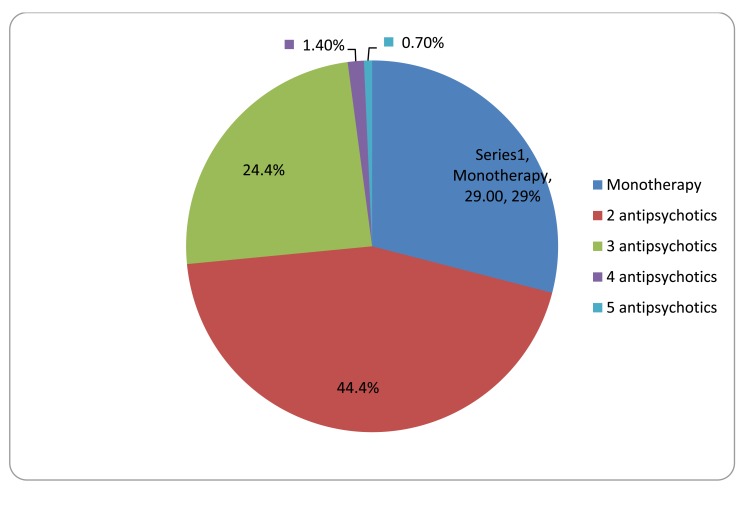
Antipsychotic use patterns of patients with schizophrenia.

The patients were evaluated according to the kinds of psychotropic medications and the pills that they used daily. The patients with schizophrenia used 2.0 ± 0.81 antipsychotics daily and 2.61 ± 1.07 psychotropic drugs (0–7). They used 3.52 ± 2.55 pills per day (1–18). Their rate of using two or more kinds of psychotropic drugs was 70.71%. The number of daily pills was 2.64 ± 2.61 for patients with monotherapy and 3.89 ± 2.46 for patients with polypharmacy (p < 0.05). The number of different kinds of antipsychotic use is given in (Table **2** and Fig. **1**).

The overall rate for the use of at least one second generation antipsychotic in all of the schizophrenic patients was 96.9% and the rate for the use of at least one typical antipsychotic was 17.2%. The rate of use of long-term antipsychotics (depot) was 56.8%.

When equivalent dosages of antipsychotics lower than 100 mg of chlorpromazine were excluded, the polypharmacy rate decreased to 63.4%, the use of two kinds of antipsychotics was 40.9%, three kinds was 21.5% and the rate for four antipsychotics was 1.1%.

**Fig. (2):**
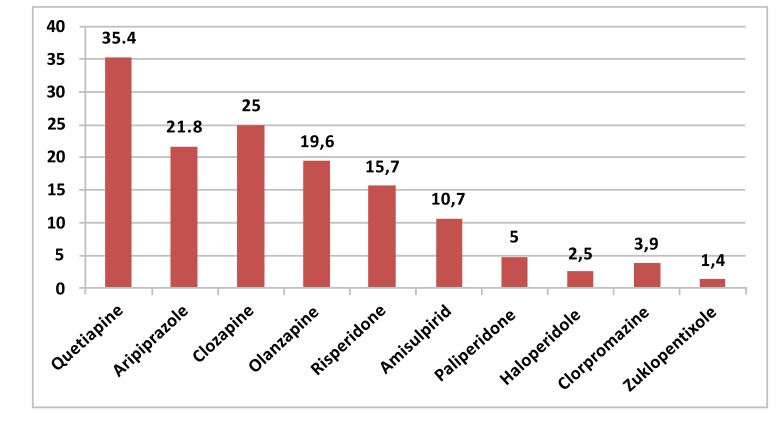
Oral forms of antipsychotics actively used by schizophrenia patients.

The most frequently used second generation antipsychotic was quetiapine, with a rate of 35.5%, and the most frequently used typical antipsychotic was haloperidol, with a rate of 2.5%. The distribution of the rates of antipsychotics is provided in (Fig. **2**).

Either anticholinergic drugs or biperiden was used by 22.9% of the patients and 11.8% of the schizophrenia patients used antidepressants. Other drugs such as benzodiazepines were used at a rate of 11.4%.

### Correlates of Polypharmacy

Using monotherapy or polypharmacy did not have a relationship with the patient’s age, gender, education, marital or occupational status. In addition, it was not related to the number of hospitalizations, the total duration of the hospital stays, having a suicidal attempt or having physical violence (all p > 0.05).

**Having a psychotic patient among the patient’s relatives** was related to polypharmacy; the patients who had a psychotic patient relative had higher rates of monotherapy than the ones who did not have a psychotic patient relative (monotherapy rate 39.4% vs 25.8%, respectively).

A daily or weekly visit with the CCS was also related with polypharmacy. The patients who regularly visited the CCS had higher polypharmacy rates than the ones who did not (polypharmacy rate 75.5% vs 64.8%, respectively).

### Clinical Severity and Functionality of the Patient and Polypharmacy

Patients with higher polypharmacy rates had a significantly higher severity of disease according to CGI-S and lower functionality according to GAF (Table **3**).

### Adherence to Treatment

The question ‘Is there nonadherence to medication during the last month?’ was answered as yes by 18.5% of the monotherapy group and 24.2% of polypharmacy group. The difference was not statistically significant (p > 0.05).

**Use of clozapine and other antipsychotics:** There was no significant relationship between the use or dosage of clozapine and polypharmacy (p > 0.05 in X^2^ for use and independent *t*-tests for dose). With the exception of quetiapine, there was no difference between the use and dose of other antipsychotics or polypharmacy.

For quetiapine, the mean dose was 105.00 ± 71.58 for monotherapy patients and 390.69 ± 261.97 for polypharmacy patients; patients who used polypharmacy also used significantly higher doses of quetiapine (p < 0.05).

**Use of depot antipsychotics:** In the polypharmacy group, the use of depot antipsychotics was 68.68% and in the monotherapy group, it was 18.86%. The polypharmacy group used depot antipsychotics in significantly higher rates (p < 0.05).

### Predictors of Polypharmacy

A regression model consisting of the associated factors was found by our results to determine the independent predictors of polypharmacy. Using a depot antipsychotic, having regular visits to the CCS (daily or weekly), clinical severity, level of functionality, having a psychotic relative, and gender (due to data at previous studies) were selected as predictors and polypharmacy was selected as the independent variable. In this model, using a depot antipsychotic, having regular visits to the CCS (daily or weekly) and clinical severity were significant (adjusted R^2^: 0.189; p < 0.05) predictors for polypharmacy.

### Mean Dosages of Antipsychotics

The mean dosages of antipsychotics were compared between monotherapy and the polypharmacy groups. The mean dosages and the final total chlorpromazine equivalent doses are given in (Tables **4** and **5**).

## DISCUSSION

This study was conducted with clinically stable schizophrenia patients who showed high rates of antipsychotic polypharmacy, at a rate of almost 71%. When the kinds of antipsychotics are corrected according to lower dosages of the CPZ-equ 100 mg, the polypharmacy (antipsychotic combination) rate was 63.2%.

According to the data in this study, the rates for polypharmacy are considered high because more than half of the patients use two or more than two antipsychotics. This is not an expected condition. In previous studies, several reasons have been shown for polypharmacy, the severity of illness, avoiding adverse effects, sociodemographic properties, including genetic and environmental influences, shifting to another drug by titrating and the treatment of adverse effects (as an adjunctive therapy) [25, 26]

High rates in Turkey may be related to many reasons. Refractory schzophrenia is shown to be related to high rates of polipharmacy and high rates of clozapine use. [27]. Our sample mostly consisted of patients who did not have enough functionality to have their own occupation and sought rehabilitation service from community counseling centers. So the probable high rates of refractory schzophrenia may be a reason.

In search for other reasoning behind polypharmacy, we considered the geographic-cultural position of the country too. In previous studies, a relationship was found between ethnicity and polypharmacy. This may be due to genetic influences on response to treatments, symptom profile of schzophrenia and all other probable related pharmacogenetics [27, 28]. Turkey is a localisation country between Asia and Europe and it has various ethnic combinations. The polypharmacy rate seems higher in this study than in the European and American studies and may be accepted as either similar with/or a bit lower than that of the Asian and other countries. The ethnic, genetic, environmental and cultural components may have roles in the formation of antipsychotic prescriptions.

In a previous study conducted in Turkey, the rate for the use of two or more antipsychotics was 40% [29]. In another study conducted by Özalmete *et al.*. (2003), the polypharmacy rate reported in hospitalized schizophrenic patients was 48.89% [30]. In 2010, a Turkish study reported the polypharmacy rate in inpatients as 72.1%; here, the psychosis group had the highest polypharmacy use among inpatients [31].

 A study in 2015 reported polypharmacy rates of 62% for schizophrenia patients [32]. Although there are methodological differences between the studies, our study is very similar to that one, which was conducted in the same country in the same year. On examining this literature, it appears that the rate of polypharmacy is increasing slightly in Turkey. Since this is a similar condition to that of other countries such as United Kingdom and South Korea, the tendency to polypharmacy has been increasing recently [33-36].

 Decrease in polypharmacy has been reported in Japan. Although there are methodological diffrences between studies, importance of health policies for decreasing polipharmacy is underlined in the study and we think on smilar pattern with that [37]. The increase in polypharmacy rates is a dynamic process so it needs explanation more dynamic than etnicity and refractory schizophrenia. For example, changes in health policies may be related to changes in the rates of polypahrmacy as in Japan.

Because we questioned whether the higher numbers belonged exclusively to the type of drugs or if the dosages were also higher, we evaluated chlorpromazine equivalent dosages of the daily antipsychotic medications. According to our study, the mean CPZeq dose in the patients was 684 mg. A Norwegian study reported the CPZeq dose at discharge as 450 mg (SD 347, range 25–2800). Another study conducted with acute schizophrenia patients reported a CPZeq dose of 942.1 mg (SD 805.6) [38]. In an East Asian study, the polypharmacy group had a higher CPZeq mean dose than the monotherapy group (983.10 vs 411.47) [7]. These results are similar to those of our study (834.32 vs 328.55).

Shifting to another antipsychotic by slow titration is a typical reason for polypharmacy [39]. However, in our study, patients with an antipsychotic combination used almost three fold higher dosages of antipsychotics than the monotherapy users. This suggests that they are combination of high doses of antipsychotics and these particular cases are not in a titration period. On the other hand, the mean dosage in polypharmacy is 831.32 mg, which is still just between the ranges of the effective dosage for chlorpromazine, albeit very near the upper limit [40]. This result suggests that practitioners do not prefer using one type of medicine in a maximum dosage but instead preferring to use the optimal dosage and another one if needed.

When they were calculated individually, except for quetiapine, there was no significant difference between the dosages of antipsychotics in the polypharmacy and monotherapy groups. This means that the same dosage of antipsychotics was used in the polypharmacy group and another one was added in the same dosage to the first one. This result also suggests a combination of optimal dosages of antipsychotics instead of the minimal dosages. This condition suggests a need for higher dosages of antipsychotics as a referee to clinical severity.

The rates and dosages of clozapine were similar in both groups. Only quetiapine was higher in the polypharmacy groups, suggesting the need for higher dosages in the polypharmacy group. This need may be related to the clinical severity of the illness. In previous studies, the clinical severity of the patients has been shown to be the greatest potential predictor parameter for polypharmacy [32]. In this study, clinical severity was related to polypharmacy as an independent predictor; clinically more severe and low functional patients used antipsychotic combinations instead of monotherapy.

The use of depot antipsychotics was more frequent in the polypharmacy group of this study, confirming the previous results [6, 41, 42]. The use of depot antipsychotics was also an independent predictor for polypharmacy in this study. Depot antipsychotics are generally preferred in patients who are not expected to adhere strictly to their treatment. Lack of insight and poor cognitive function are central to poor treatment adherence in schizophrenia [43].

 Perhaps it is speculative, since low-treatment adherence has been reported in previous studies, but we anticipated that the use of depot antipsychotics might refer to a clinically difficult situation to manage [6]. So, depot antipsychotics may be the reason of polypharmacy but a special group of patients with low adherence to treatment or treatment resistance may be reasons for both the need of depot antypsyhotics and polypharmacy. Therefore, polypharmacy may be sometimes considered in association with a clinical need.

Polypharmacy results in an increased number of daily pills. In this study, the mean daily pill count was 3.52 and it increased up to 18 pills daily. Although the polypharmacy group in this study used a significantly higher number of daily pills, there was no significant difference between the monotherapy and the polypharmacy group. This result was congruent with a previous study that showed that treatment adherence was not directly related to the number of medications but adverse effects increase with polypharmacy in patients with schizophrenia [44].

In this study, there was a relationship between monotherapy and having a relative with schizophrenia. This may have multiple explanations. Knowing the disease may help a better experienced patient care and strict follow up of medicines and this may help decrease polypharmacy with a better response to treatment on monothraphy. But knowing the disease may be related also to having realistic expectations about remission, in this situation, a lower degree of enforcement for a better clinical condition may result in monotherapy.

 Probably the patients, who have been seen In congruence with this hypothesis (high expectations: more drugs), 'visiting a community counselling centre regularly' had higher polypharmacy rates; furthermore, it was an independent predictor in regression analysis. We considered that the CCS team realized the symptoms of the patients by regular/frequent visits and this caused higher degrees of enforcement to remission of symptoms; therefore, more drugs were used for a better clinical condition. This data is interesting because we think it has a reference to symptoms focused treatment of schizophrenia. Kind and dosage of medication are managed according to the symptoms.

 Probably the patients, who have been seen more frequently by the doctor, had more opportunity to tell about their symptoms or the symptoms were observed by the medical team more than others, and the medical team needed to treat these symptoms with more medications than usual (others). Probably, we need to discuss the appropriate approach while managing the treatment of schizophrenia, as a cluster of symptoms or as an integrative syndrome includes daily, social, cognitive functionality, symptoms and treatment adherence, adverse effects *etc*.

This study showed that the second generation of antipsychotics was most widely used in schizophrenia outpatients and the most commonly used agent was quetiapine. According to another previous study conducted with outpatients in Turkey, the most common antipsychotic use was depot neuroleptics 46.7%, risperidone 45.0% and olanzapine 45.0% [29]. A recently conducted study in the US reported that risperidone was the most frequently used antipsychotic agent and the most frequent combination polypharmacy included FGA, olanzapine and risperidone. The most preferred antipsychotic combination was quetiapine plus risperidone [12]. In Germany, it was olanzapine [45] and in Korea, it was risperidone and haloperidol but the monotherapy group was olanzapine. In the UK (2007–2011), the primary care setting most commonly prescribed first- and second-generation antipsychotics: olanzapine, risperidone and chlorpromazine, respectively [46]. In Spain, olanzapine and risperidone were reported [11]. However, quetiapine is the most prescribed antipsychotic for off-label use in general mental disorders by psychiatrists in New Zealand and physicians in the US [47, 48]. Although quetiapine was the most commonly prescribed antipsychotic by psychiatrists for schizophrenia in our study, almost all of it was used in the polypharmacy group. It appears that it is prescribed as an antipsychotic agent and for other symptoms such as depression, insomnia, anxiety, *etc*.

This study investigated the current status of antipsychotic use patterns of outpatients with schizophrenia. Although the small sample size is a limitation of the study, it presents data from two large cities in Turkey. Adverse effects of antipsychotics, clozapine plazma level, symptom and functional outcome of the patients were not assessed as other limitations. The strengths of the study are that experienced psychiatrists confirmed the diagnoses of the patients and the uses of the drugs were evaluated by face-to-face interviews instead of retrospective prescription records.

## CONCLUSION

This study showed that polypharmacy is used in high rates in Turkey; claiming a pattern of clinical need, second generation antipsychotics are used in very high rates and quetiapine is the most frequently used antipsychotic. The clinical severity of the patient is an important factor for polypharmacy. Regular visits to CCS were related to higher polypharmacy rates. High rates of polypharmacy require detailed explanations along with its rationale and risk factors. Studies assessing physician- health policy related factors should be conducted as well as patient related factors. Thus, creating preventative health policies may be realistic and possible.

## Figures and Tables

**Table 1:** Sociodemographic and clinical properties of the patients.

			Female	Male	Total
			N (%)	N (%)	N (%)
Gender			94 (33.6%)	186 (66.4%)	280 (100%)
Marital status	Single		58 (61.7%)	127 (68.1%)	185 (65.9%)
	Married		16 (17.0%)	39 (21.1%)	55 (19.7%)
	Divorced-widowed *etc*		20 (21.3%)	20 (10.8%)	40 (14.4%)
Occupational status	Occupied		29 (30.8%)	79 (42.7%)	108 (38.7%)
	No occupation		42 (43.6%)	54 (28.6%)	95 (33.7%)
	Retired		5 (5.3%)	17 (9.2%)	22 (7.9%)
	Governmental support		19 (20.0%)	36 (19.4%)	55 (19.7%)
Community counselling centre visits		Daily or weekly	54 (57.4%)	101 (54.6%)	155 (55.6%)
		Irregular or rare	40 (42.6%)	85 (45.4%)	125 (44.4%)
Violence behaviour		Yes	33 (35.1%)	71 (38.4%)	104 (37.3%)
		No	61 (64.9%)	114 (61.6%)	175 (62.7%)
Suicidal attempt		Yes	31 (33.0%)	46 (24.9%)	77 (27.6%)
		No	64 (67.0%)	139 (75.1%)	202 (72.4%)
Age (mean±sd)			41.37±10.67	40.71±9.53	40.93±9.91
Years of education (mean±sd)			7.53±3.98	8.74±3.59	8.33±3.77
Duration of illness (years) (mean±sd)			17.86±9.44	16.82±9.06	17.17±9.18
Number of hospitalizations (mean±sd)			2.32±2.64	3.14±3.12	2.87±2.99
Total stay in hospital (days)(mean±sd)			66.72±107.28	89.40±104.39	81.81±105.69

**Table 2:** Number of antipsychotics used.

	Total	
Use of antipsychotic	N	(%)
Monotherapy	82	29.28%
Polypharmacy	198	70.71%
2 antipsychotics	124	44.4%
3 antipsychotics	68	24.4%
4 antipsychotics	4	1.4%
5 antipsychotics	2	0.7%

**Table 3:** Clinical severity and functionality of the patient and polypharmacy.

		Monotherapy(N=82)	Polypharmacy(N=198)	Total(N=280)	p	Z
CGI-S	Mean±Std Dev	3.84±0.838	4.21±0.852	4.10±0.863	0.000	-3.687
GAF	Mean±Std Dev	55.90±9.964	52.94±10.192	53.81±10.197	0.012	-2.525

CGI-S: Clinical global impression score GAF: General Assessment of Functionality Score

**Table 4:** Mean daily dosages of chlorpromazine equivalent doses.

		Monotherapy	Polypharmacy	Total
Female (N=94)	Mean±SD	288.9±152.5	788.2±388.32	627.5±408.7
	Min–Max	32.5–594.0	65.0–1636.3	32.50–1636.33
Male (N=186)	Mean±SD	357.0±191.9	852.4±455.1	713.1±456.8
	Min–Max	46.67–900.0	122.3–2670.7	46.67–2670.7
Total (N=280)	Mean±SD	328.55±183.4	834.32±441.0	684.08±442.18
	Min–Max	32.5–900.0	65.0–2670.7	32.5–2670.7

**Table 5:** Comparison of mean daily dose of antipsychotics between polypharmacy and monotherapy groups.

	Monotherapy		Polypharmacy	
	Mean(mg)	SD(mg)	Mean(mg)	SD(mg)
Clozapine	392.50	207.919	368.37	188.108
Quetiapine	105.00	71.589	390.69	261.971
Olanzapine	13.80	6.668	14.35	6.027
Risperidone	5.20	4.604	3.61	1.830
Paliperidone	7.00	1.732	7.29	3.604
Aripiprazole	15.83	8.010	19.14	9.801
Amisulpride	600.00	282.843	596.43	244.165
Haloperidol	20.00	0	8.86	4.180
Clopixol	50.00	0	26.67	20.817
Chlorpromazine	0	0	113.64	63.604
